# Diacylglycerol triggers Rim101 pathway–dependent necrosis in yeast: a model for lipotoxicity

**DOI:** 10.1038/s41418-017-0014-2

**Published:** 2017-12-11

**Authors:** Patrick Rockenfeller, Martin Smolnig, Jutta Diessl, Mina Bashir, Vera Schmiedhofer, Oskar Knittelfelder, Julia Ring, Joakim Franz, Ines Foessl, Muhammad J. Khan, René Rost, Wolfgang F. Graier, Guido Kroemer, Andreas Zimmermann, Didac Carmona-Gutierrez, Tobias Eisenberg, Sabrina Büttner, Stephan J. Sigrist, Ronald P. Kühnlein, Sepp D. Kohlwein, Campbell W. Gourlay, Frank Madeo

**Affiliations:** 1 Institute of Molecular Biosciences, NAWI Graz, University of Graz, Graz, 8010 Austria; 2 Kent Fungal Group, School of Biosciences, University of Kent, Canterbury, CT2 7NJ UK; 3 Department of Molecular Biosciences, The Wenner-Gren Institute, Stockholm University, Svante Arrheniusväg 20C, Stockholm, 106 91 Sweden; 4 Division of Endocrinology and Diabetology, Medical University of Graz, Graz, 8010 Austria; 5 Max Planck Institute of Molecular Cell Biology and Genetics, Dresden, 01307 Germany; 6 Institute of Molecular Biology and Biochemistry, Medical University of Graz, Graz, 8010 Austria; 7 INSERM U848, Villejuif, 94805 France; 8 Metabolomics Platform, Institut Gustave Roussy, Paris, 94805 France; 9 Centre de Recherche des Cordeliers, Paris, 75006 France; 10 Pôle de Biologie, Hôpital Européen Georges Pompidou, Paris, 75015 France; 11 Université Paris Descartes, Sorbonne Paris Cité, Paris, 75270 France; 12 BioTechMed-Graz, Graz, 8010 Austria; 13 Institute for Biology, Freie Universität Berlin, Berlin, 14195 Germany; 14 NeuroCure Charité, Berlin, 10117 Germany; 15 Max Planck Institute for Biophysical Chemistry, Göttingen, 37077 Germany; 16 Present Address: Department of Biosciences, COMSATS Institute of Information Technology, Park Road, Islamabad, 44000 Pakistan

## Abstract

The loss of lipid homeostasis can lead to lipid overload and is associated with a variety of disease states. However, little is known as to how the disruption of lipid regulation or lipid overload affects cell survival. In this study we investigated how excess diacylglycerol (DG), a cardinal metabolite suspected to mediate lipotoxicity, compromises the survival of yeast cells. We reveal that increased DG achieved by either genetic manipulation or pharmacological administration of 1,2-dioctanoyl-*sn*-glycerol (DOG) triggers necrotic cell death. The toxic effects of DG are linked to glucose metabolism and require a functional Rim101 signaling cascade involving the Rim21-dependent sensing complex and the activation of a calpain-like protease. The Rim101 cascade is an established pathway that triggers a transcriptional response to alkaline or lipid stress. We propose that the Rim101 pathway senses DG-induced lipid perturbation and conducts a signaling response that either facilitates cellular adaptation or triggers lipotoxic cell death. Using established models of lipotoxicity, i.e., high-fat diet in *Drosophila* and palmitic acid administration in cultured human endothelial cells, we present evidence that the core mechanism underlying this calpain-dependent lipotoxic cell death pathway is phylogenetically conserved.

## Introduction

The maintenance of precise lipid compositions is crucial to guarantee membrane integrity, proper signaling, and trafficking. Most eukaryotic organelle membranes consist of phosphatidylcholine (PC), phosphatidylethanolamine, phosphatidylserine, phosphatidylinositol, phosphatidic acid (PA), diacylglycerol (DG), sterols, and sphingolipids. These lipids differ in their characteristics regarding bilayer formation, curvature determination, regulation of fission, and fusion processes, and membrane protein embedding [1]. How cells regulate and maintain the lipid composition of membranes is not yet fully understood but is a crucial requirement to facilitate their diverse functions.

Lipid overload can lead to cellular lipotoxicity, which in higher eukaryotes can trigger tissue degeneration, precipitating a number of diseases, including metabolic syndrome, type II diabetes mellitus, cardiovascular disorders, hepatosteatosis, and cancer [2, 3]. The lipid species which are most relevant for lipotoxicity are under discussion, but most probably include free fatty acids (FFA), ceramide, cholesterol, and DG [3–6]. Although evidence for the lipotoxic nature of these lipids exists, the exact mechanisms underlying lipotoxic cell death remain unclear [7].

DG is a central intermediate in the synthesis of membrane phospholipids and the storage lipid, triacylglycerol (TG), and its cellular steady state levels are typically very low. De-regulated DG levels, on the other hand, are suspected to be involved in the development of insulin resistance and diabetes [8], and its abundance correlates with the occurrence of non-alcoholic fatty liver disease, including steatosis, steatohepatitis and cirrhosis [6]. An inherent problem of these studies, however, is that the regulation of DG takes place at multiple anabolic and catabolic levels and in various subcellular compartments. Given that, experimental manipulation of DG concentrations is an extremely difficult task. The different DG pools within subcellular compartments such as the endoplasmic reticulum (ER), lipid droplets or plasma membrane, their metabolic origins (TG synthesis, TG lipolysis, and phospholipid turnover) and regio isomerism (*sn*-1,2-, *sn*-1,3-, or *sn*-2,3-) of DG need to be considered for its biological activity and metabolic fate [9]. Given the experimental restrictions in higher eukaryotes, we decided to study DG toxicity in the budding yeast *Saccharomyces cerevisiae*, since its genetics offer the unique possibility to cut off all DG-catabolizing pathways. Using two different strategies to accumulate DG in yeast, we found that increased levels of DG are sufficient to induce cell death and identified glucose repression and Rim101 signaling as crucial mediators of DG lipotoxicity. Additional experimental evidence obtained using established lipotoxicity models in *Drosophila* and a human endothelial cell line suggest that the core of this lipotoxicity pathway is evolutionary conserved in metazoans.

## Results

### A genetically engineered yeast strain accumulates DG

**Fig. 1:**
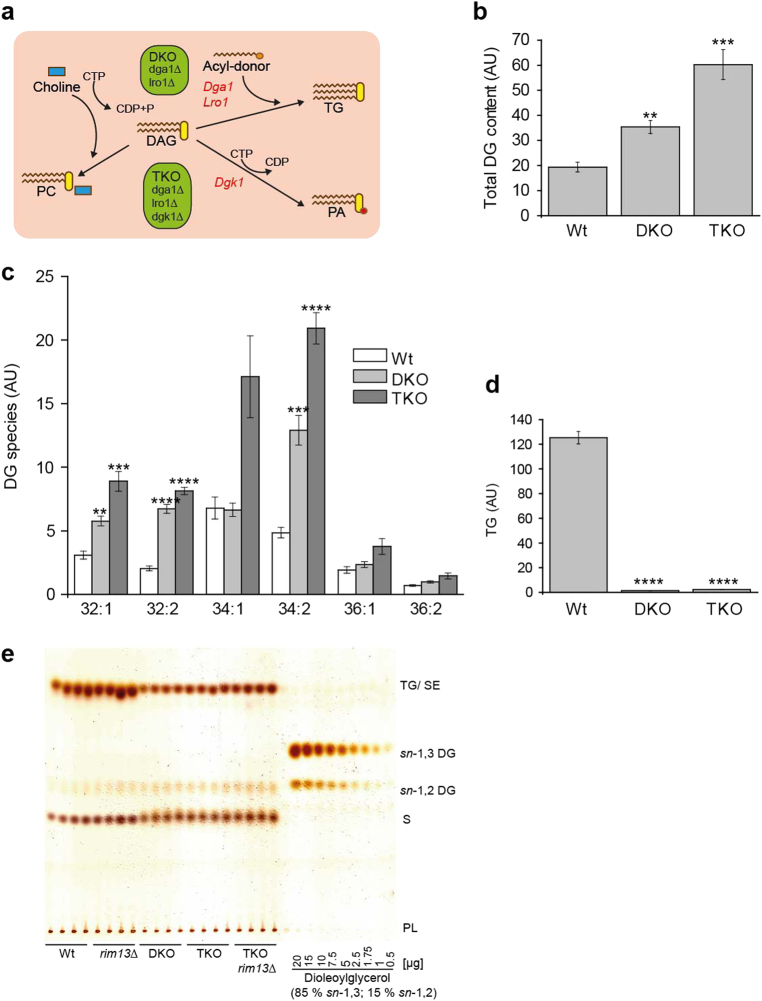
Lipidomic characterization of a *dga1∆ lro1∆ dgk1∆* triple knockout strain (TKO) reveals a huge increase in diacylglycerol (DG) levels **a** Schematic illustration of the pathways that lead to DG accumulation in the *dga1∆ lro1∆* DKO and *dga1∆ lro1∆ dgk1∆* TKO strains: DG is either transformed into triacylglycerol (TG) by acylation with activated fatty acids (acyl-CoA) or acyl-residues derived from phospholipids through Dga1 or Lro1, respectively, or may be phosphorylated to phosphatidic acid (PA) by the action of Dgk1. The DKO (*dga1*∆ *lro1*∆) mutant is defective for TG formation and therefore accumulates DG. The additional knock out of *DGK1* encoding DG kinase, in the TKO strain further increases DG accumulation. Administration of choline directly drains DG into phosphatidylcholine (PC) through the Kennedy pathway and thus facilitates growth of the TKO mutant. **b**–**d** Mass spectrometry-assisted quantification of lipids from total yeast cell extracts harvested 12 h after inoculation: total DG (**b**), DG species (**c**), and total TG (**d**). The numbers on the *x* axis of **c** indicate the cumulative number of carbon atoms (first number) and the number of double bonds in both acyl-chains (second number after the colon) **e** Thin layer chromatography performed with the same lipid extracts as were used for MS analysis. Comparison to the standard allows to differentiate between *sn-*1,3 and *sn-*1,2 DG species. SE steryl esters, S sterols, PL phospholipids. Statistical significance was assessed using one-way ANOVA for **b** and **d** and *T*-Test for **c**. Error bars indicate SEM and asterisks in the figures indicate significant differences, *p < 0.05, **p < 0.01, ***p < 0.001, ****p < 0.0001

To increase cellular DG levels, we generated an *S. cerevisiae* triple knockout strain (TKO), which accumulates endogenous DG. This was achieved by deleting genes of three DG-metabolizing enzymes: (i) *DGA1*, (ii) *LRO1*—both encoding DG-acyl transferases catalyzing the last step of TG formation—and (iii) *DGK1*, responsible for the phosphorylation of DG to PA (Fig. 1a). A third major DG-consuming reaction via the Kennedy pathway was modulated by the addition (or omission) of choline [10], which drains DG into the synthesis of PC (Fig. 1a).

Mass spectrometric lipid analysis revealed that, as expected, the TKO (*dga1*∆ *lro1*∆ *dgk1*∆) contained increased intracellular DG levels (Fig. 1b, c), while levels of TG (Fig. 1d) were decreased. Interestingly, a double-knockout (DKO) mutant deleted for *DGA1* and *LRO1* genes also displayed a moderate but significant increase in DG (Fig. 1b, c) allowing us to comparatively analyze different DG levels by using either the DKO or the TKO strains. Thin layer chromatography revealed that the accumulating DG species had *sn*-1,2 configuration, consistent with their origin from de novo synthesis (Fig. 1e).

### DG accumulation leads to increased necrotic cell death and ROS production

**Fig. 2:**
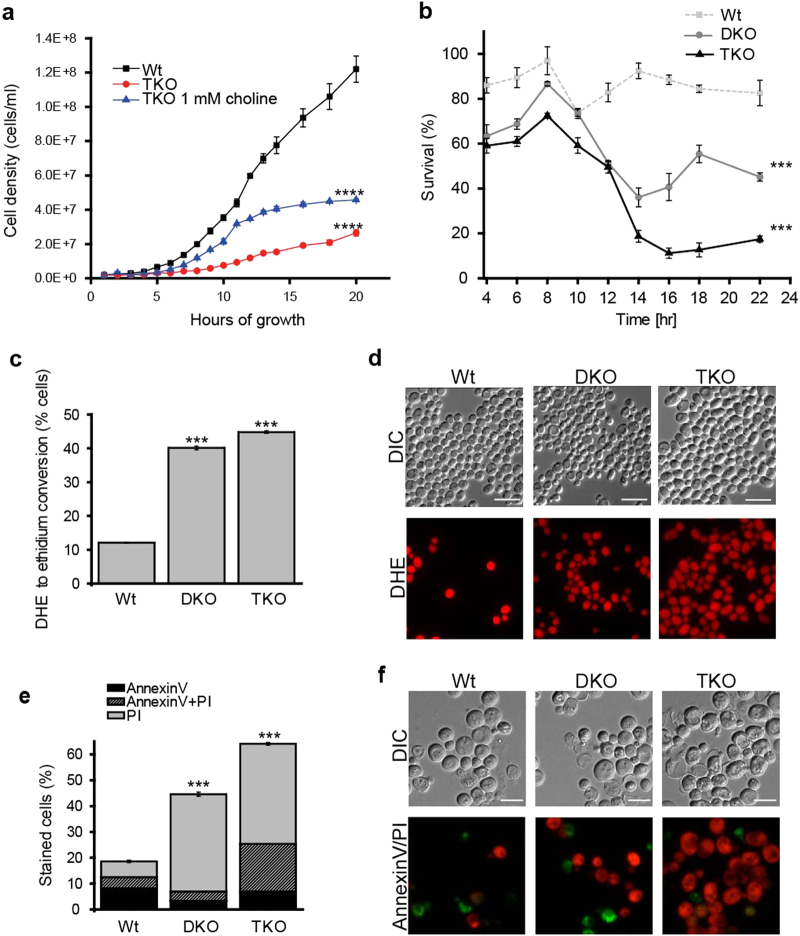
Endogenous DG accumulation triggers ROS production and necrosis **a** Growth curve of the TKO in comparison to wild type (Wt). Administration of 1 mM choline chloride increases the growth rate of the TKO. **b** Time course survival assay based on clonogenicity of yeast cells. **c** Flow cytometry-assisted analysis of DHE to ethidium conversion for ROS quantification measured 20 h after inoculation. **d** Microscopy pictures of the same samples used in **c**. Scale bar = 10 µm. **e** Flow cytometry-assisted analysis of AnnexinV (green)/ PI (red) co-staining for differentiation between apoptosis and necrosis measured 20 h after inoculation. p-values were calculated for the PI positive fractions. **f** Representative microscopy images of the same samples as used in **e**. Scale bar = 5 µm. For panels a and b statistical significance was assessed using two-way ANOVA with time and strain as independent factors. Panel c and d were analyzed using one-way ANOVA. ANOVA analysis in d refers to the PI-only-positive fraction shown in gray. Error bars indicate SEM and asterisks in the figures indicate significant differences, *p < 0.05, **p < 0.01, ***p < 0.001, ****p < 0.0001

The toxic effects of DG accumulation could be observed by a significant decrease in growth in the TKO strain that was partly restored by administration of 1 mM choline chloride (Fig. 2a). A time course monitoring clonogenic survival upon DG accumulation showed that the severity of inflicted lipotoxicity was proportional to DG levels (Fig. 2b). This reduction in survival was accompanied by the overproduction of reactive oxygen species (ROS), as determined by the ROS/superoxide-dependent conversion of non-fluorescent dihydroethidium (DHE) to fluorescent ethidium (Fig. 2c, d). As ROS production often precedes programmed cell death we made use of an AnnexinV/PI assay to differentiate between apoptotic and necrotic cell death [11]. In this assay, apoptosis is represented by the AnnexinV-positive fraction, whereas secondary necrosis and primary necrosis are represented by the AnnexinV/PI-double-positive or PI-only-positive fractions, respectively [11, 12]. The PI-only-positive fraction was predominant in DKO and TKO cells suggesting a necrotic type of cell death (Fig. 2e, f).

**Fig. 3:**
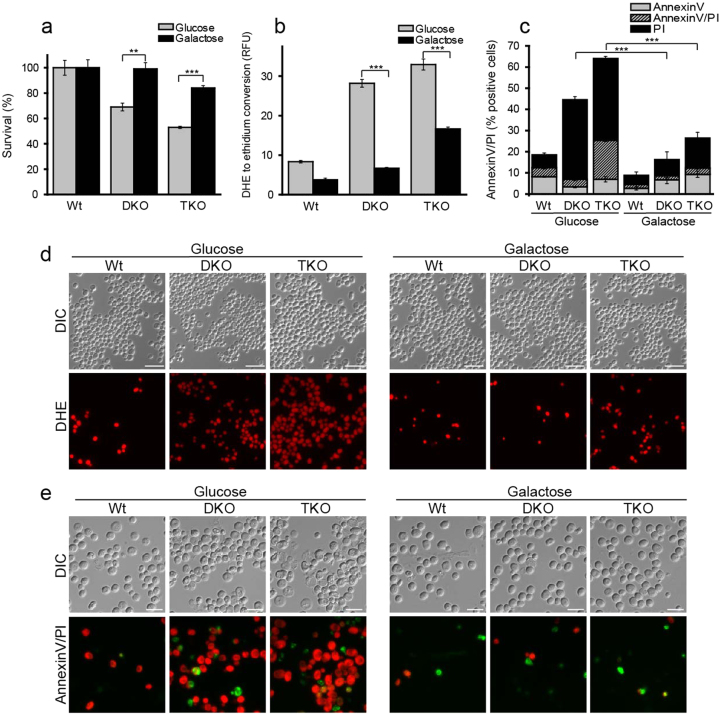
ROS accumulation and cell death depend on glucose as carbon source Clonogenic survival assay **a** and ROS assessment **b** comparing DKO and TKO cells to wild type (Wt) using glucose or galactose as carbon source. Flow cytometry-assisted analysis of AnnexinV/PI co-staining **c** of the same samples used in panels a and b for differentiation between apoptosis and necrosis. p-values are calculated for the PI positive fractions. Representative microscopy images of ROS assessment **d** shown in panel b. Scale bar = 15 µm. Representative images of AnnexinV/PI-based cell death assay **e** as shown in panel c. Scale bar = 7.5 µm. All measurements were made at 20 h after inoculation. Panels a-c were analyzed using one-way ANOVA. ANOVA analysis in d refers to the PI-only-positive fraction shown in black. Error bars indicate standard error of the mean (SEM) and asterisks in the figures indicate significant differences, *p < 0.05, **p < 0.01, ***p < 0.001, ****p < 0.0001

We next compared the survival of DKO, TKO and wild-type strains using different carbon sources. Interestingly, cell death was only triggered upon growth on glucose-containing media (Fig. 3a) while ROS accumulation and necrotic cell death were significantly reduced when glucose was replaced with galactose (Fig. 3b–e). In summary, our data suggest that an increase in cellular DG induces glucose-dependent and ROS-associated necrotic cell death in yeast.

### Administration of a small, cell permeable DG analog triggers glucose-dependent necrosis in yeast

**Fig. 4:**
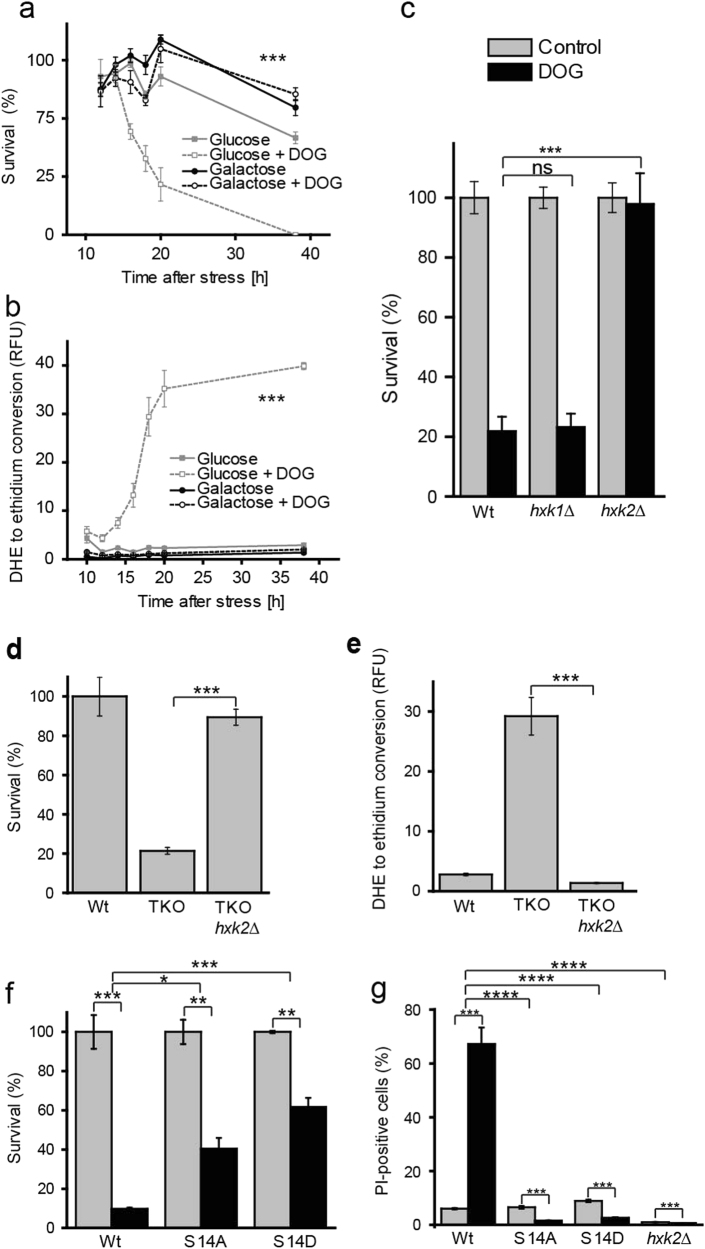
DG-mediated cell death depends on glucose and hexokinase 2 mediated glucose repression The time course of clonogenic survival **a** and ROS production **b** of wild-type yeast grown on glucose or galactose as carbon sources was determined with and without DOG administration. **c** Clonogenic survival assay of hexokinase deletion mutants (*hxk1∆* and *hxk2∆*) with and without DOG treatment. Clonogenic survival assay **d** and ROS assessment **e** comparing TKO *hxk2∆* to TKO and wild-type (Wt) cells. **f** Clonogenic survival plating and **g** PI-measurement of *hxk2* mutants. S14D represents the phospho-mimetic mutant, whereas mutant S14A is not subject to phosphorylation at position 14 anymore. Statistical significance in panels a and b was assessed using three-way ANOVA with carbon source, treatment and time as independent factors. **c**, **f** and **g** were analyzed using two-way ANOVA with strain and treatment as independent factors. Error bars indicate SEM and asterisks in the figures indicate significant differences, *p < 0.05, **p < 0.01, ***p < 0.001, ****p < 0.0001

We made use of the small, cell permeable DG analog 1,2-dioctanoyl-*sn*-glycerol (DOG) as a complementary approach to increase cellular DG in yeast. DOG administration has been previously used to mimic endogenous DG in the fission yeast *Schizosaccharomyces pombe* [13] and in mammalian cells [14] for investigating both protein kinase C-dependent and independent roles of DG. Importantly, external DOG administration to wild-type yeast cultures led to the induction of cell death (Fig. 4a), which was accompanied by the accumulation of ROS (Fig. 4b). In order to test whether the production of ROS was causally linked to cell death induction, we made use of the ROS scavenger N-acetyl cysteine [15], which we administered to the yeast cultures. Our results reveal that ROS scavenging only shows limited potential in preventing cell death in both our model systems of DAG-induced cell death (Supplementary Fig. 1a, b). Interestingly, the effects of DOG treatment were limited to cells cultured in glucose medium as growth on galactose (Fig. 4a, b) and raffinose (data not shown) entirely prevented DOG-induced cell death and ROS accumulation. ROS accumulation and cell death were detectable after 14 h of DOG treatment when cells usually begin to exhaust glucose and enter the diauxic shift phase of growth. We hypothesized that the metabolic changes that occur due to glucose depletion would be responsible for cell death induction by DOG. We therefore conducted a series of experiments, in which we shifted DOG-exposed cells onto spent media. Indeed, exposure to spent media led to a rapid decrease in survival (Supplementary Fig. 1c) that was attributable to necrotic cell death (Supplementary Fig. 1d). This cell death could be prevented by glucose supplementation (Supplementary Fig. 1e, f).

### DOG-induced lipotoxicity depends on glucose repression and de-repression

Glucose repression has been extensively studied in yeast and is known to regulate a number of genes [16]. We conducted a screen to investigate whether glucose repression is actively involved in DOG-mediated cell death (Supplementary Fig. 1g). Intriguingly, we found that the master regulator of glucose repression, hexokinase 2 (Hxk2*)*, is a crucial factor for DOG-mediated cell death, whereas hexokinase 1, which is not involved in glucose repression, is dispensable for this effect (Fig. 4e). Of note, *HXK2* deletion in the TKO background resulted in a similar outcome in that survival was significantly increased whereas ROS accumulation was substantially decreased (Fig. 4f, g).

The regulatory function of Hxk2 with respect to glucose repression can be separated from its enzymatic activity as hexokinase [17]. Phosphorylation of Hxk2 at serine 14 regulates its localization to either the cytoplasm or nucleus, and determines its role in glucose repression [18]. To assess whether abrogation of the regulatory function of Hxk2 was sufficient to prevent cell death, we made use of two Hxk2 mutants, one in which serine 14 residue was replaced by alanine (S14A) and another in which serine 14 was changed to aspartate (S14D). The non-phosphorylatable Hxk2^S14A^ mutant is found in the cytoplasm and nucleus and constitutively activates glucose repression, while the phospho-mimetic Hxk2^S14D^ is localized to the cytosol and fails to activate glucose repression. In line with our hypothesis, expression of Hxk2^S14D^ suppressed DOG-induced cell death (Fig. 4f, g). However, the rescue of cell death was unexpected for the S14A mutation, as our initial hypothesis pictured glucose repression as being crucial and committing for cell death induction. The results from the S14A experiment rather suggested that constant glucose repression was not sufficient to trigger cell death. This led us to conclude that sequential de-repression after glucose repression is required for DOG-induced cell death.

### DOG-induced cell death depends on the calpain-like cysteine protease Rim13

**Fig. 5:**
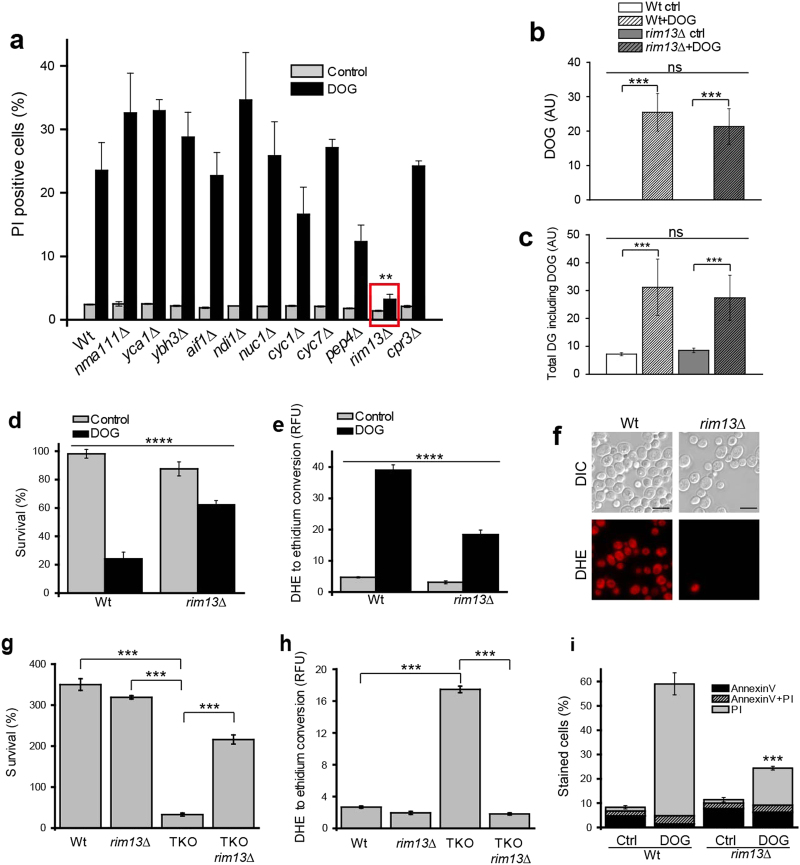
DG induces Rim13-dependent necrosis **a** Screen for knockout mutants rescuing DOG-induced cell death based on PI staining. **b**,** c** Mass spectrometry-assisted quantification of DOG **b** and total DG **c** levels. **d** Clonogenic survival plating and **e** DHE to ethidium conversion for ROS quantification after DOG treatment comparing wild type to *rim13∆*. **f** Representative microscopy pictures of ROS assessment for DOG-treated samples. Scale bar = 5 µm. Clonogenic survival assay **g** and ROS assessment **h** comparing TKO *rim13∆* to DKO, TKO and wild-type cells. **i** FACS-assisted analysis of AnnexinV/PI co-staining. Statistical significance in panels a-d was analyzed using two-way ANOVA with strain and treatment as independent factors. Panels b and c were additionally analyzed by one-way ANOVA to assess significant change after DOG treatment. Statistical significance of **g**, **h**, and **i** was determined using one-way ANOVA. Significance in i refers to the PI fraction. Error bars indicate standard error of the mean (SEM) and asterisks in the figures indicate significant differences, *p < 0.05, **p < 0.01, ***p < 0.001, ****p < 0.0001

In order to further understand the mechanistic details of how yeast cells die in response to DG, we applied DOG treatment to all deletion mutants of known and putative yeast programmed cell death (PCD) regulators [19–23]. This screen revealed that the yeast calpain-like protease Rim13 (alternatively called Cpl1) is essential for DOG-mediated lipotoxicity (Fig. 5a). Cells lacking Rim13 were completely resistant to DOG-mediated toxicity, as determined by PI staining (Fig. 5a). Importantly, the deletion of known regulators of yeast apoptosis such as *YCA1* [20] and *AIF1* [22] did not influence the DOG-induced cellular demise. Mass spectrometric analysis confirmed that administered DOG readily entered the yeast cells, intracellular DG levels were not affected by *RIM13* deletion and DOG treatment substantially increased total cellular DG levels (Fig. 5b, c, Supplementary Fig. 2a).

Deletion of *RIM13* also significantly reduced ROS accumulation and cell death rates upon DOG treatment (Fig. 5d–f, Supplementary Fig. 2b, c). Deletion of *RIM13* in the TKO background led to increased survival (Fig. 5g) and reduced ROS accumulation (Fig. 5h), further suggesting that *RIM13* is crucial for triggering DG-induced cell death. Importantly, *RIM13* deletion did not affect lipid levels in the TKO strain (Supplementary Fig. 3). To discriminate between apoptosis and necrosis in the DOG treatment setting, we performed an AnnexinV/PI assay. The increase in necrotic cells that is usually observed when DOG was added to wild-type yeast cells was lost upon deletion of *RIM13* (Fig. 5i, Supplementary Fig. 4).

### DOG-triggered PCD requires Rim13 cysteine protease activity, its carboxy-terminal domain and depends on Rim101 processing

**Fig. 6:**
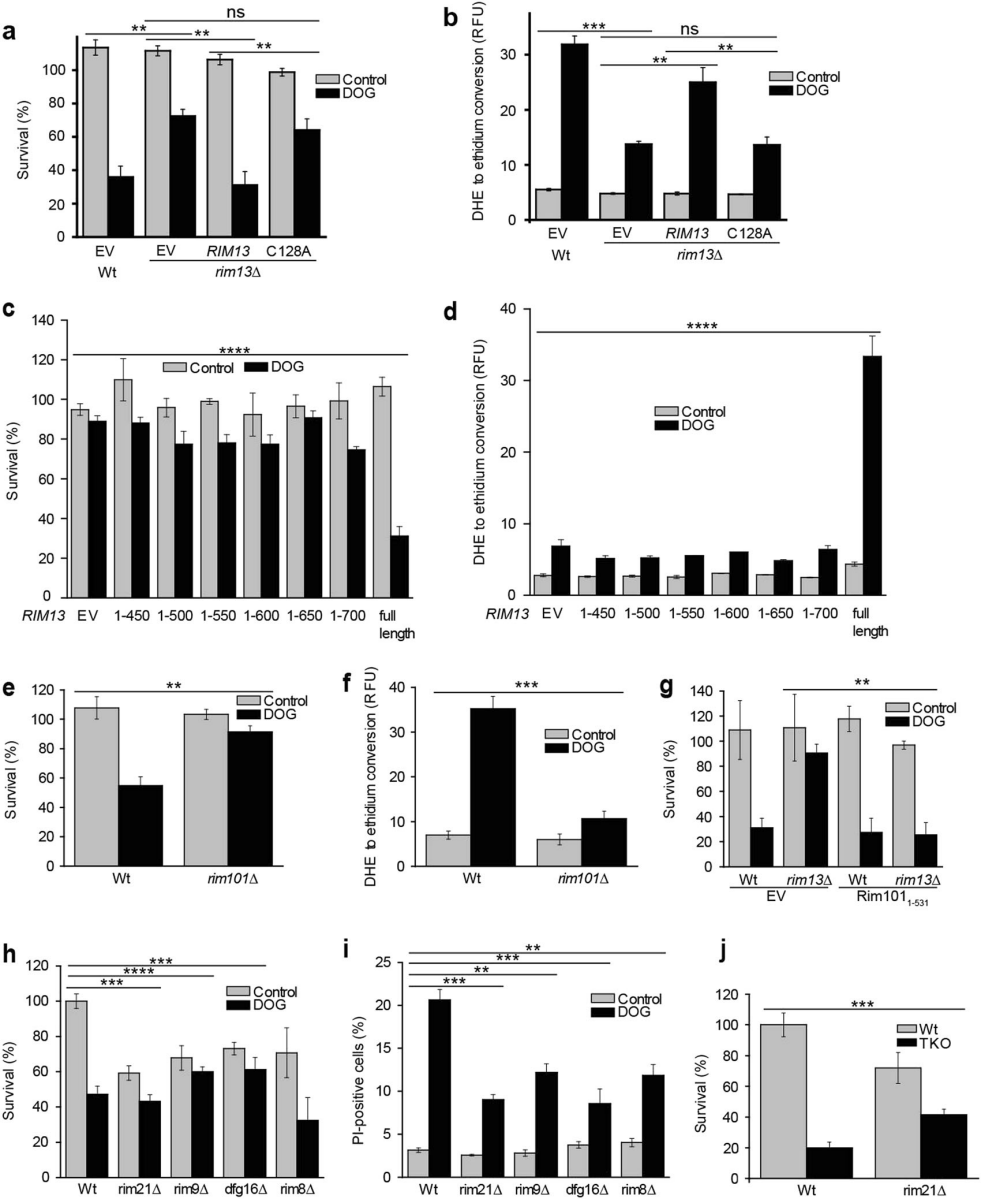
The Rim101 cascade is involved in DOG-induced cell death **a** Clonogenic survival and **b** DHE to ethidium conversion of *rim13∆* upon DOG treatment expressing either the wild-type *RIM13* gene or *rim13* bearing the C128A point mutation. DHE to ethidium conversion was detected 24 h and survival was determined 25 h after DOG stress. **c** Clonogenic survival and **d** DHE to ethidium conversion upon DOG treatment was detected in *rim13∆* with expression of carboxy-terminally truncated or full *RIM13* on a plasmid. **e** Clonogenic survival and **f** DHE to ethidium conversion of *rim101∆* upon DOG treatment. **g** Clonogenic survival of *rim13∆* with and without expression of constitutively active Rim101_1-531_ upon DOG treatment. ‘EV’ indicates empty vector control. **h** Clonogenic survival assay and **i** PI staining were detected in Rim21 sensor complex single deletion mutants *rim21*∆, *rim9*∆, *dfg16*∆, and *rim8*∆ upon DOG stress **j** Clonogenic survival assay and the impact of *RIM21* deletion in TKO background. PI staining of the same experiment is shown in Supplementary Fig. 5a. Statistical significance was assessed using two-way ANOVA with strain and treatment as independent factors. Error bars indicate SEM and asterisks in the figures indicate significant differences, *p < 0.05, **p < 0.01, ***p < 0.001, ****p < 0.0001

Next we attempted to complement the lack of *RIM13* in the knock out strain by expression of *RIM13* on a plasmid that is under control of its endogenous promoter. To verify whether cell death depends on the proteolytic activity of Rim13, a point mutation in which cysteine_128_ of the active site was exchanged for alanine was also generated. As expected, the DOG-resistance of the *rim13*∆ strain was reverted when *RIM13* was reintroduced (Fig. 6a). ROS production was also restored to wild-type levels in the *rim13∆* strain upon re-expressing *RIM13* (Fig. 6b). This rescue was not observed upon replacement of endogenous *RIM13* by the *rim13* ^*C128A*^ mutant allele, indicating that the cysteine protease function of Rim13 is crucial for cell death (Fig. 6a, b). A similar complementation assay in the *rim13*∆ strain revealed that its carboxy-terminal domain is also required for cell death (Fig. 6c, d). This domain has previously been linked to the regulation of Rim13 localization and Rim13 mediated cleavage of the transcriptional repressor Rim101 [24]. *RIM101* deletion itself was also protective against DOG-mediated cell death induction (Fig. 6e) and ROS accumulation (Fig. 6f). Importantly, expression of a carboxy-terminally truncated Rim101 version (Rim101_1-531_), which is constitutively active [25], re-established DOG-mediated cell death in the *rim13∆* strain (Fig. 6g). Rim101 activation through manipulation of plasma membrane lipids has been reported to depend on the Rim21 sensor complex which includes *RIM21*, *RIM9*, *DFG16*, and *RIM8* [26, 27]. This raised the question whether DG-induced cell death would also depend on this sensor complex. We addressed this question in such a way that we subjected all the single knock outs of the sensor complex (*rim21*∆, *rim9*∆, *dfg16*∆, and *rim8*∆) to DOG treatment and assessed the survival by clonogenic survival assay and PI staining. Our results reveal that the sensor complex is indeed crucial to fully conduct the lipotoxic response (Fig. 6h, i). To further investigate this in our genetic model of DG increase, we knocked out *RIM21* in the TKO background and measured the impact on survival. The obtained data (Fig. 6j and Supplementary Fig. 5a) confirm that Rim21 also has a functional role in this setting of DG-induced cell death suggesting that DG-mediated activation of the Rim101 pathway actively involves the Rim21 sensor complex.

In summary, our yeast data provide evidence for the existence of a lipotoxic cell death pathway that can be triggered by excess DG (Supplementary Fig. 5). This pathway is dependent on functional glucose repression and de-repression and requires Rim13-mediated activation of the transcriptional repressor Rim101.

### The calpain-dependent lipotoxic cell death pathway is evolutionary conserved

**Fig. 7:**
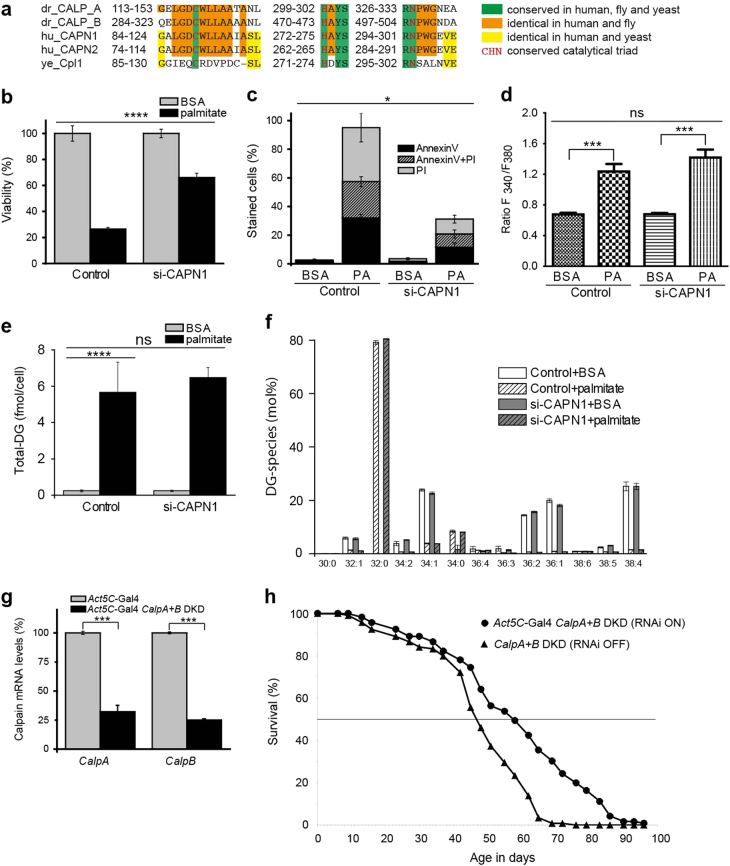
Calpain-dependent lipotoxic cell death is evolutionary conserved **a** Alignment of calpain protein sequences from different organisms: *Drosophila CalpA* and *B* (dr_CALPA/B), human calpain 1 and 2 (huCALP1/2), and the yeast calpain-like protease (yeCpl1/Rim13). **b** The viability of palmitate-treated endothelial cells was determined using the MTT assay. **c** Quantification of AnnexinV/ PI positive cells from microscopy images. **d** Quantification of basal cytosolic Ca^2+^ in response to palmitate treatment in endothelial cells **e**,** f** Shotgun mass spectrometry-assisted quantification of total DG **e** and DG species **f** from endothelial cells. The full lipidomics data set is shown in Supplementary Fig. 6b. **g** Quantitative real time PCR was performed to assess mRNA levels of *CalpA* and *CalpB* in whole *Drosophila*. Relative mRNA levels were calculated in comparison to control flies and normalized to the ribosomal protein *RpL32*. **h** Ubiquitous *CalpA*+*B* double gene knockdown (DKD) confers lifespan extension of *Drosophila melanogaster* on high-fat diet. Representative lifespan experiment showing median and maximum lifespan extensions of *Act5C*-Gal4 *CalpA*+*B* DKD flies (57/84 days) compared to control (47/63 days) male flies by 21% (median) and 33% (maximum), respectively. Lines represent mean age-specific survivorship data of *n* = 120 males per genotype. Survivorship data of the two genotypes are significantly different (log-rank test *p* < 0.001). Statistical significance in panels b–e was assessed using two-way ANOVA with siRNA-mediated knockdown and palmitate treatment as independent factors. Data in panels d and e were additionally processed by one-way ANOVA to assess significance of palmitate treatment. Statistical significance in panel g was determined using one-way ANOVA. Error bars indicate SEM and asterisks in the figures indicate significant differences, *p < 0.05, **p < 0.01, ***p < 0.001, ****p < 0.0001

To investigate whether the lipotoxic cell death pathway that we identified in yeast is evolutionarily conserved across metazoa, we decided to test two well-established models of lipotoxicity in higher organisms: palmitic acid stress applied to mammalian cell culture and coconut oil rich high-fat diet (HFD) fed *Drosophila melanogaster*. Importantly the central regulator of lipotoxic cell death that we have identified in yeast, Rim13, is phylogenetically conserved (Fig. 7a). Palmitic acid increases cytosolic Ca^2+^ levels and induces programmed necrosis in endothelial cells [28]. Small interfering RNA (siRNA)-mediated knockdown of m-calpain-1 (CAPN1), the human orthologue of *RIM13* (Fig. S6a) rendered cells resistant to palmitic acid stress (Fig. 7b, c), as evidenced by increased viability (Fig. 7b) and reduction of cell death markers (Fig. 7c). Importantly the levels of basal cytosolic Ca^2+^ were not affected upon CAPN1 knockdown (Fig. 7d). Palmitic acid stress has been described to increase DG levels in H9C2 cardiomyoblasts [29] which we could confirm in a lipidomic approach using our endothelial cell line (Fig. 7e, Supplementary Fig. 6b). Further analysis of the DG species revealed that the total DG increase is mostly due to palmitate incorporation into DG as documented by the 32:0 species, which harbors two palmitate residues (Fig. 7f). Interestingly, DG accumulation was not dependent on calpain 1 as no reversion of this phenomenon was observed with CAPN1 knockdown cells (Fig. 7e, f). We thus suggest that palmitic acid-induced cell death is mediated through DG accumulation and that calpain acts downstream of this event.

Feeding a coconut-based HFD to *Drosophila* leads to adverse effects such as body fat increase, reduced activity, cardiomyopathy and a reduced lifespan [30, 31]. *Drosophila* has four *Calp* genes, two of which (*CalpA* and *CalpB*) encode proteolytically active calpains [32]. We thus generated a ubiquitous *CalpA*+*B* double gene knockdown (*CalpA*+*B* DKD) and compared the lifespans on HFD with the RNAi turned on or off. Importantly, reduction of *CalpA* and *CalpB* expression to 25% of the normal level (Fig. 7g) increased the median lifespan on HFD by 21% and the maximum lifespan by 33% (Fig. 7h). The bodyweight of DKD flies was not reduced with respect to controls, excluding a potential dietary restriction-like effect (Supplementary Fig. 6c). In a nutshell, these findings suggest that calpains act as key regulators for organismal lipotoxicity.

## Discussion

In fungi the Rim101 pathway constitutes a well conserved signal transduction route that is primarily known for sensing and reacting to pH alteration [33, 34]. Importantly, it is required for the pathogenicity of *Candida albicans*, *Cryptococcus* species and other noxious fungi [35]. However, the Rim101 pathway not only senses alkaline conditions, but also recognizes lipid alterations in the plasma membrane such as changes in the asymmetrical lipid distribution among the two leaflets of the bilayer [26, 36, 37]. The Rim101 pathway includes a sophisticated sensor complex consisting of Rim8 and the three transmembrane proteins Dfg16, Rim9 and Rim21. The carboxy-terminal cytosolic domain of Rim21 localizes to the plasma membrane under normal conditions, whereas lipid perturbation of the plasma membrane triggers its dissociation [38]. The sensor complex then induces a downstream proteolytical complex, which consists of Rim13, Rim20, Ygr122w, and Rim101 [33]. The cysteine protease Rim13 is the sole yeast orthologue of mammalian calpains, explaining its alternative denomination as Cpl1 (Calpain-like protease) [39]. Rim13 proteolytically cleaves and thereby activates the transcriptional repressor Rim101 [39]. Most processes of the Rim101 cascade have been studied as a response to pH stress. Induction of the Rim101 pathway as a result of lipid stress at the plasma membrane has been investigated by means of genetic deletion of flippases and floppases or their upstream regulators [26, 38, 40], by addition of palmitoleic acid [41] and by expressing phospholipase A2 [42]. In most of these studies, it appears that Rim21 senses changes in the plasma membrane´s lipid asymmetry in a similar way as under alkaline conditions, since both conditions interfere with the charge gradient present at the plasma membrane. Hence, disturbance of specific physicochemical properties of the plasma membrane could be perceived by the sensing complex involving Rim21 under both conditions of stress. Carboxy-terminal cleavage of Rim101 by Rim13 uncovers its gene repressing activity. A number of Rim101-repressed genes have been identified by the Mitchell lab so far and include *NRG1*, *PRB1*, *RIM8*, *SMP1*, *YJR061W*, *YOR389W*, and *YPL277C*, the promoters of which directly interact with Rim101 [43]. Nrg1 and Smp1 themselves represent transcription factors on a second level and contribute to the large complexity of Rim101 controlled gene regulation.

Activation of the Rim101 pathway is the natural response to alkaline or lipid stress in yeast that is presumably activated with the scope of rearranging the lipid composition of the plasma membrane and hence to facilitate adaptation to environmental changes. Here we have shown that, in the case of non-physiological elevation of DG, this particular stress response is triggering a necrotic type of lipotoxic cell death rather than an adaptive response (Figs. 2, 5). Our results reveal that two transcriptional regulatory pathways (i) glucose repression and (ii) the Rim101 pathway including its Rim21-dependent sensing complex are involved in DG-mediated lipotoxicity. This raised the question whether these two pathways interfere with cell death regulation on the same level, culminating in a similar outcome or whether they represent distinct routes. Interestingly, the transcriptional repressor Nrg1 lies at the crossroads of glucose repression and the Rim101 pathway and could thus represent such a downstream regulator integrating signals from these different routes. Nrg1 controls repression of the *SUC2* and *GAL* genes, which are needed for growth on alternative carbon sources, as well as for haploid invasive growth and diploid pseudohyphal differentiation [44]. Nrg1 further represses the expression of the sphingoid long-chain base efflux transporter Rsb1 [45, 46]. Rsb1 in turn upregulates Lem3 which controls the flippases Dnf1 and Dnf2 and negatively regulates the floppase Yor1, possibly explaining the lipid stress-induced changes of the plasma membrane [41]. Smp1, the other transcriptional repressor which is regulated by Rim101, controls rough colony morphology, sporulation, and haploid invasive growth [43]. Altogether, this suggests that excess DG activates the Rim101 pathway that integrates its signal with the glucose repression pathway on the level of transcriptional repressors and thereby regulates cell fate decisions. We hypothesize that these decisions can include adaptation to the initial stress or the initiation of regulated cell death [47].

The production of ROS could potentially be regulated by glucose repression and Rim101 signaling, both pathways of which merge at the level of *NRG1*. Nrg1 further regulates the repression of genes involved in alternative carbon source usage as mentioned above [16]. This might actually affect respiration and electron transport efficiency within respiratory complexes and thus impact ROS production. However, since the effects of ROS scavenging cannot fully account for an essential role in DG-induced necrosis (Supplementary Fig. 1a, b) we rather think that ROS accumulate as a matter of altered lipid metabolism or as part of a failed cellular attempt to prevent cell death. The fact that DG-induced cell death depends on glucose repression and de-repression highlights that the metabolic state of cells is important to make them susceptible to cell death induction. After the diauxic shift, when toxic effects are starting to become visible, the cells have an increased demand for lipid mobilization for example to feed into mitochondrial membrane expansion to allow for efficient respiration. This could explain for the sudden toxicity of DG arising after the metabolic shift. Further research will need to be conducted to fully answer these interesting questions.

Intriguingly, calpains have been identified as crucial regulators of necrotic cell death in diverse models of neurodegeneration [48, 49]. The lipotoxic models we used in Fig. 7 suggest that calpain-mediated lipotoxic cell death might have developed rather early during evolution and that its core function as a regulator of lipotoxic cell death is conserved across species. Calpain-mediated lipotoxicity could further be important for lipid-associated disease or during aging and neurodegeneration, where metabolic changes are also prevalent. Calpain might have evolved as a regulator of lipid metabolism to sense and react to changes in the lipid environment. Our lipotoxic models are suitable to further investigate the underlying mechanism which will be addressed in further studies.

## Methods

### Yeast strains and growth conditions

All experiments were carried out in the BY4741 (*MAT* ***a*** *his3∆1 leu2∆0 met15∆0 ura3∆0*) strain background. Single deletion strains were obtained from the EUROSCARF knock out collection except for the self-generated *rim13∆* strains in Supplementary Fig. 1. The *dga1∆ lro1∆* DKO was generated in a previous study [50]. The *dga1∆ lro1∆ dgk1∆* TKO was generated in the DKO background by using the *URA3* gene disruption cassette, which was subsequently excised by Cre-recombinase expression to regain the *ura3* marker [51]. The same technique was used to generate four independent *cpl1∆* strains, which all yielded the same phenotypes as the EUROSCARF *rim13∆* strain. Strains were grown in SC medium containing 0.17% yeast nitrogen base (Difco), 0.5% (NH_4_)_2_SO_4_ and 30 mg/l of all amino acids (except 80 mg/l histidine and 200 mg/l leucine), 30 mg/l adenine, and 320 mg/l uracil with 2% glucose as carbon source for SCD medium or 2% galactose for SCG medium, respectively. All yeast cultures were inoculated from a stationary overnight culture to an OD_600_ = 0.1 and then grown at 28 °C and 145 rpm shaking for indicated time periods. For DOG stress experiments, cultures were stressed with 1.45 mM DOG (1,2-dioctanoyl-*sn*-glycerol, Cayman) at an OD_600_ = 0.35. For ROS scavenging experiments N-acetylcysteine (NAC) was added to a final concentration of 10, 30 and 100 mM at 14 h after DOG stress or 14 h after inoculation for TKO experiments.

### Cloning and molecular biology

*RIM13/CPL1* was cloned into plasmid pRS313 under its endogenous promoter using the *NotI* and *EcoRI* restriction sites. The C128A mutation was introduced using two-step PCR assisted site-directed mutagenesis [52]. The oligonucleotides used for cloning are listed in Supplementary Table 1.

### Survival plating and test for apoptotic/necrotic markers

For survival plating, the cell concentrations of culture dilutions were determined with a CASY cell counter (Schaerfe Systems) and aliquots containing 500 cells were plated on YPD plates. The number of colonies formed was determined after 2 days at 28 °C. AnnexinV/PI co-staining was performed as previously described [11]. 30,000 cells were evaluated using flow cytometry and analyzed using BD FACSDiva software. For dihydroethidium staining, 5 × 10^6^ cells were harvested by centrifugation, resuspended in 250 μl of 2.5 μg/ml DHE in PBS, and incubated in the dark for 5 min. Relative fluorescence units (RFU) were determined using a fluorescence reader (Tecan, GeniusPRO) and then normalized to an OD_600_ of 0.2. For ROS analysis based on individual cells flow cytometry was used to count positive cells. The same samples were analyzed by fluorescence microscopy on a Zeiss Axioskop microscope equipped with a rhodamine filter set.

### Lipid analysis and mass spectrometry in yeast

After addition of 50 µl of an internal standard mix (Supplementary Table 2) to each sample, total lipids were extracted from exponentially growing yeast cultures (harvested 12 h after inoculation) with chloroform/methanol 2:1 (v/v) according to Folch et al. [53]. The organic phase was dried under a stream of nitrogen, and dissolved in 500 μl of chloroform/methanol (2:1, v/v).

Analysis of lipid extracts was carried out by an ACQUITY-UPLC system (Waters, Manchester, UK) equipped with a BEH C18 column (2.1 × 150 mm, 1.7 µm, Waters) coupled to a SYNAPT G1 qTOF HD mass spectrometer (Waters) [54]. Separation of lipids was achieved with a binary gradient consisting of solvent A water/methanol (1:1, v/v) and solvent B isopropanol. Each solvent contained 8 µM phosphoric acid, 10 mM ammonium acetate and 0.1 Vol% formic acid. The gradient started at 45% solvent B and was increased to 90% B within 30 min. Thereafter, the eluent was raised to 100% B within 2 min and kept for another 10 min. The system was changed back to starting conditions within 1 min, and the column was equilibrated for 7 min prior to the next analysis. The column was kept at 50 °C in a column oven. Ionization was achieved by an ESI source using the following parameters: capillary temperature: 100 °C, desolvatization temperature: 400 °C, N_2_ as nebulizer gas. The capillary voltage was set to 2.1 kV in negative mode and 2.6 kV in positive mode. Injection of the sample ranged from 5–10 µl depending on the used ionization mode (pos/neg). For positive mode the samples were diluted 1:5 with isopropanol whereas for negative mode samples were dissolved in a mixture of isopropanol and chloroform/methanol 2:1 (9:1, v/v) and injected without prior dilution. Leucine enkephaline (Sigma-Aldrich) at a concentration of 100 pg/µl in water/acetonitrile (1:1, v/v)+0.1 Vol% formic acid as a lock mass reference was supplied by an external pump (L-6200 intelligent pump, Hitachi) at a flow rate of 0.2 ml/min splitted in a 1:13 ratio. For data acquisition MSE scan mode was applied to generate full- and MS/MS scans. Data were analyzed by the MassLynx 4.1 (Waters) and by “Lipid Data Analyzer” software [55].

Neutral lipid separation and analysis was performed by thin layer chromatography (TLC) on silica gel plates (Merck), essentially as described before [56], using chloroform/acetone/acetic acid (90:8:1, per vol) as solvent. TLC plates were dipped into 3.2% H_2_SO_4_ and 0.5% MnCl_2_ followed by carbonization at 120 °C for 30 min and photometric scanning in a CAMAG TLC scanner.

### Cell culture, annexinV/PI staining and siRNA-mediated gene silencing

Endothelial cells from the human umbilical vein endothelial cell-derived cell line EA.hy926 were used in this study. Cells were grown in DMEM containing 10% FCS, 1% HAT (5 mM hypoxanthin, 20 μM aminopterin and 0.8 mM thymidine), 50 units/ml penicillin, 50 μg/ml streptomycin at 37 °C in 5% CO_2_ atmosphere.

For microscopic analysis endothelial cells grown on glass coverslips were transfected with calpain 1 (CAPN1; siRNA from Qiagen (SI02757314)) or scrambled control siRNA at 60 percent confluence and were treated with 0.5 mM palmitic acid complexed to bovine serum albumin (BSA) in a ratio of 6:1 for 16 h or BSA (control) after 48 h of transfection. Cells were stained with AnnexinV-Fluos staining kit from Roche Biodiagnostics (Roche Diagnostics GmbH). According to the manufacturers protocol 20 μl of AnnexinV-Fluos were diluted in 1 ml of incubation buffer and 20 μl of propidium iodide (PI) was added. 100 μl of this mixture were added directly to the culture and cells were analyzed after 20 min of incubation.

High-resolution imaging of AnnexinV/ PI was performed using an array confocal laser scanning microscope (ACLSM) as described previously [28] using a 40× oil objective. Cells were selected randomly on wide field and then excited with 488 and 515 nm simultaneously for AnnexinV and PI fluorescence, respectively. Images were captured by a charged-coupled device (CCD) camera (CoolSNAP-HQ, Photometrics, Tucson, USA). All devices were controlled by VisiView Premier acquisition software (Visitron Systems, Puchheim, Germany). Cytosolic Ca^2+^ measurement: For cytosolic Ca^2+^ measurements the Fura-2 technique was used as described previously [28].

### Lipid extraction from endothelial cells and quantification by shotgun mass spectrometry

Cell pellets were homogenized with 1 mm zirconia beads in a cooled tissuelyzer for 2 × 5 min at 30 Hz in 300 µl IPA. An aliquot of the homogenate was used for protein determination by BCA assay and 50 µg of total protein were used for lipid extraction [57, 58]. In brief, 700 µl internal standard mix in 10:3 methyl tert-butyl ether/methanol was added to each sample and vortexed for 1 h at 4 °C. After addition of 140 µl H_2_O samples were vortexed for another 15 min. Phase separation was induced by centrifugation at 13,400 r.p.m. for 15 min. The organic phase was transferred to a glass vial and evaporated. Samples were reconstituted in 300 µl methanol/chloroform (1/2 per vol). For lipidome and PS measurements 5 µl of sample were diluted with 95 µl isopropoanol/ methanol/chloroform (4/2/1 per vol)+7.5 mM ammonium formate and ethanol/chloroform (4/1 per vol)+0.1% trimethylamine, respectively.

Mass spectrometric analysis was performed on a Q Exactive instrument (Thermo Fisher Scientific, Bremen, DE) equipped with a robotic nanoflow ion source TriVersa NanoMate (Advion BioSciences, Ithaca, USA) using nanoelectrospray chips with a diameter of 4.1 μm. The ion source was controlled by the Chipsoft 8.3.1 software (Advion BioSciences). Ionization voltage was +0.96 kV in positive and −0.96 kV in negative mode; back pressure was set at 1.25 psi in both modes. Samples were analyzed by polarity switching [58]. The temperature of the ion transfer capillary was 200 °C; S-lens RF level was set to 50%. Each sample was analyzed for 5.7 min. FT-MS spectra were acquired within the range of m/z 400–1000 from 0 min to 1.5 min in positive and within the range of m/z 350–1000 from 4.2 min to 5.7 min in negative mode at a mass resolution of R m/z 200 = 140,000, automated gain control (AGC) of 3 × 10^6^ and with an maximal injection time of 3000 ms. PS was additionally measured for 1.5 min in neg FT-MS mode with the same parameters as mentioned above. All acquired spectra were filtered by PeakStrainer (https://git.mpi-cbg.de/labShevchenko/PeakStrainer/wikis/home) [59]. Lipids were identified by LipidXplorer software [60]. Molecular Fragmentation Query Language (MFQL) queries were compiled for PC, PCO, LPC, PE, PEO, LPE, PI, LPI, Cer, PA, LPA, PG, PS, TG, DG lipid classes. The identification relied on accurately determined intact lipid masses (mass accuracy better than 5 p.p.m.) and signal to noise threshold higher than 3. Lipids were quantified by comparing the isotopically corrected abundances of their molecular ions with the abundances of internal standards of the same lipid class.

### Standards for lipid quantification

Synthetic lipid standards were purchased from Avanti Polar Lipids, Inc. (Alabaster, USA). All used solvents were of at least HPLC grade. Stocks of internal standards were stored in glass ampoules at −20 °C until used for the preparation of internal standard mix in MTBE/methanol (10/3 per vol). 700 µl internal standard mix contained: 1040 pmol cholesteryl ester 16:0 D_7_, 521 pmol 50:0 TG D_5_, 145 pmol 34:0 DG D_5_, 550 pmol 25:0 PC, 435 pmol LPC, 107 pmol 25:0 PS, 295 pmol 25:0 PE, 85 pmol 13:0 LPE, 192 pmol 25:0 PI, 109 pmol 25:0 PG, 73 pmol 30:1 Cer, 123 pmol 25:0 PA, 91 pmol 13:0 LPA, 32 pmol 13:0 LPI.

### Fly techniques

The y[1] w*; P{w[+*mC] = Act5C-*GAL4*}25FO1/CyO*, y+(short name: *Act5C*-Gal4) fly stock (BDSC #4414) is available from the Bloomington Drosophila Stock Center. The transgenic RNAi fly lines, which allow conditional gene knockdown of the *CalpA* (*P{KK104532}VIE-260B*; short name *CalpA* KD; VDRC #101294) and *CalpB* (*w[1118]; P{GD16349}v46241/TM3*; short name *CalpB* KD; VDRC #46241) genes, respectively as well as the *w[1118]* mutant control line (VDRC #60000) were acquired from the Vienna Drosophila RNAi Center. RNAi effector transgene flies, which allow *CalpA* and *CalpB* double-knockdown are represented by the fly stock w*; P{KK104532}VIE-260B; P{GD16349}v46241 / TM3 Sb*, P{w[+*] Ubx-lacZ}* (short name *CalpA*+*B* DKD). All flies were propagated at 25 °C with a 12 h:12 h light/dark cycle on a complex malt-soy flour-corn flour-molasses standard food (SD) as described before [41].

### RNA extraction and assessment of RNAi-mediated *Calp* gene knockdown

Ten flies per sample were disintegrated and homogenized followed by Trizol extraction. The homogenate was extracted once with chloroform and the RNA was precipitated form the supernatant with isopropanol and washed with 70% ethanol. 500 ng RNA were treated with DNaseI followed by reverse transcription using Superscript III Reverse Transcriptase (Invitrogen Inc.). For quantitative real time PCR Invitrogen´s SYBR Select Master Mix was used. *CalpA* and *CalpB* gene expression levels of male flies subject to ubiquitous *CalpA*+*B* DKD double knockdown (*y* ^*1*^ w*; P{w[+*mC] = Act5C-GAL4}25FO1/P{KK104532}VIE-260B; P{GD16349}v46241 /*+) were compared to controls (*y* ^*1*^ w*; P{w[+*mC] = Act5C-*GAL4*}25FO1/*+). qRT-PCR was performed using the primers listed in Table S1 and *CalpA* and *CalpB* expression was normalized to *RpL32* gene expression levels to assess the efficiency of the knock downs.

### Lifespan assay

Lifespan assays were performed as described before [41]. In brief, *n* = 120 age-matched male flies (12 replicates of cohorts of 10 flies) for each genotype were raised on SD until hatching to adult flies. Animals were kept at 23 °C with a 12 h:12 h light/dark cycle on high-fat diet (HFD) food, which was prepared by adding 30 ml coconut oil (Sigma) to 100 ml of SD. The HFD food was changed every 3–4 days and the dead flies were scored and removed from the experiment at indicated time points. Data plotted in Fig. 7f show a representative of two experiments, which displays age-specific average survivorship rate means calculated from the replicates. Maximal lifespan values correspond to 10% survivorship. A log-rank test was performed to assess statistical significance of the observed differences between the *CalpA*+*B* DKD flies and the control flies survivorship data. Average fly wet-weight was determined by weighing cohorts of 10 flies each.

### Statistical analysis

Statistical analyses were calculated in Prism7. For assessment of significance one-way, two-way or three-way ANOVA were performed as indicated. Error bars indicate SEM and asterisks in the figures indicate significant differences, *p < 0.05, **p < 0.01, ***p < 0.001, ****p < 0.0001.

## Electronic supplementary material

Supplemental data
