# Supplementary material for: Diacylglycerol triggers Rim101 pathway–dependent necrosis in yeast: a model for lipotoxicity
Source: Cell Death Differ. 2017 Dec 11;25(4):765–81. doi: 10.1038/s41418-017-0014-2 (PMC5864183; doi:10.1038/s41418-017-0014-2)
Supplement: Supplementary file 1 — Supplemental data [file 41418_2017_14_MOESM1_ESM.docx]

**Supplemental data**

**Table S1, related to materials and methods.** Primer sequences used in this study

| No. | Name | Sequence |
| --- | --- | --- |
| 301 | *CPL1*_fwd | GACCATTTCAGTCGAAGACAAGAACCTTGTGGTATCAAAACAGCTGAAGCTTCGTACGC |
| 302 | *CPL1*_bwd | AAAAAGGATGGATAGACAGTTCTTGAGATAAAGCACAGGCATAGGCCACTAGTGGATCTG |
| 424 | *DGK1*_fwd | CATTTCTGTCATTTGGATTCATACAGGGTTTACAGACAATCAGCTCAGCTGAAGCTTCGTACGC |
| 425 | *DGK 1*_bwd | GACTTACCGAAGAATATAAAACACTCCTGTTTTTGGTATATATGCGCATAGGCCACTAGTGGATCTG |
| 426 | *DGK 1*_ctrl | CAAGACATGGCTGCTAGA |
| 428 | *CPL1*_ctrl-new | CAGTGAGCTTCCTATTGTTAAAG |
| 607 | *CPL1*endprom-NotI_fw | ATCTGCGGCCGCCACTTGCTCAGTAGCTGAGGA |
| 608 | *CPL1*endprom-EcoRI_bw | ATCTGAATTCTTATTTTGGTATCACATCATCGGAG |
| 385 | pUGL_C128A_fwd | GCCGAGATGTGCCAGATgcCTCATTAGTTGCTTCTCTAATTAATC |
| 386 | pUGL_C128A_bwd | AGAAGCAACTAATGAGgcATCTGGCACATCTCGGCATTGTTC |
| 651 | CalpA_fwd | CGAGATCTTCATCAAGAAGGAGGCC |
| 652 | CalpA_rev | CCTGCACCTCGGAATAGGGTCC |
| 653 | CalpB_fwd | GCAAAGCTGCATGGATCTTATGAAGC |
| 654 | CalpB_rev | CCTCCTTGATGTCGTACCACTCG |
| 655 | RpL32_fwd | GCTAAGCTGTCGCACAAATGGCG |
| 656 | RpL32_rev | GGTTCTGCATGAGCAGGACCTC |

**Table S2.** Internal standard mix for mass spectrometry as used in yeast experiments. Phospholipids were purchased from Avanti Polar Lipids and neutral lipids (TG, DG) from Larodan.

| Lipid species | Concentration [mg/ml] |
| --- | --- |
| DG 28:0 (14:0 x 2) | 0.05 |
| TG 36:0 (12:0 x 3) | 0.05 |
| TG 45:0 (15:0 x 3) | 0.05 |
| TG 51:0 (17:0 x 3) | 0.05 |
| TG 57:0 (19:0 x 3) | 0.05 |
| PC 24:0 (12:0 x 2) | 0.05 |
| PC 34:0 (17:0 x 2) | 0.05 |
| PC 38:0 (19 x2) | 0.05 |
| PE 24:0 (12:0 x 2) | 0.05 |
| PE 34:0 (17:0 x 2) | 0.05 |
| PS 34:0 (17:0 x 2) | 0.05 |
| PA 24:0 (12:0 x 2) | 0.05 |
| PA 28:0 (14:0 x 2) | 0.05 |
| PA 34:0 (17:0 x 2) | 0.05 |


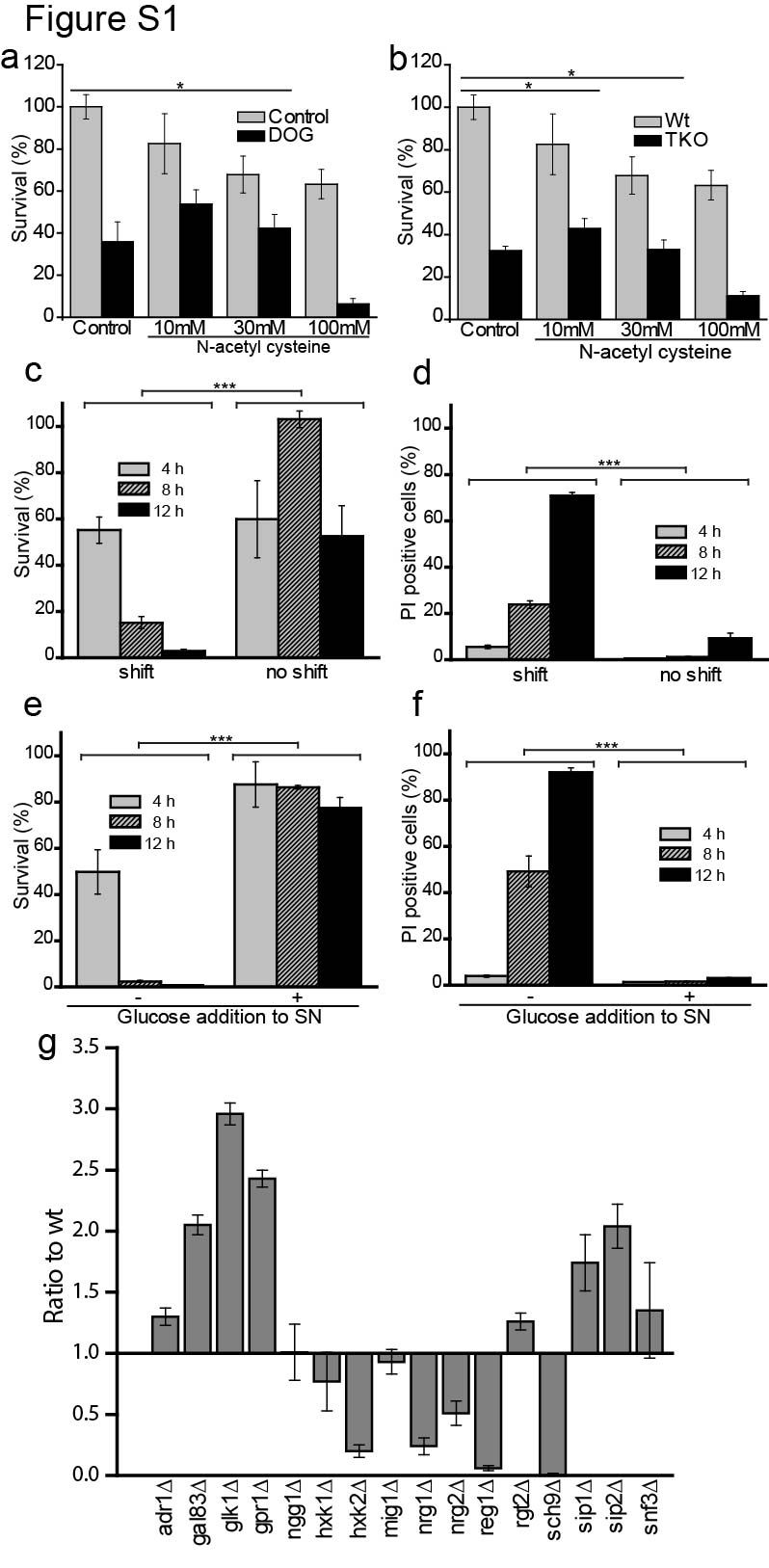


**Figure S1**, related to Fig. 4. (a, b) Clonogenic survival assays with ROS scavenging by N-acetyl cysteine (NAC) at 10, 30 and 100 mM upon DOG administration (a) or comparing TKO to wildtype (b). (c-f) Clonogenic survival data and propidium iodide (PI) quantification from shift experiments are shown, in which the supernatant (SN) of growing cultures has been exchanged for spent media. Clonogenic survival (c) and PI quantification (d) of cells shifted for 4, 8 and 12 h is shown in comparison to non-shifted cells. Glucose addition to the spent SN prevented from premature cell death induction as evidenced by clonogenic survival assay (e) and PI quantification (f). Two-way ANOVA analysis was performed with time and condition as independent factors to assess statistical significance. (g) Screen for potential modulators of DOG-induced cell death using deletion mutants of non-essential glucose repression-related genes. PI-positivity was used as read out and data are presented as fold changes compared to wild type. Error bars indicate standard error of the mean (SEM) and asterisks in the figures indicate significant differences, *p<0.05, **p<0.01, ***p<0.001, ****p<0.0001.


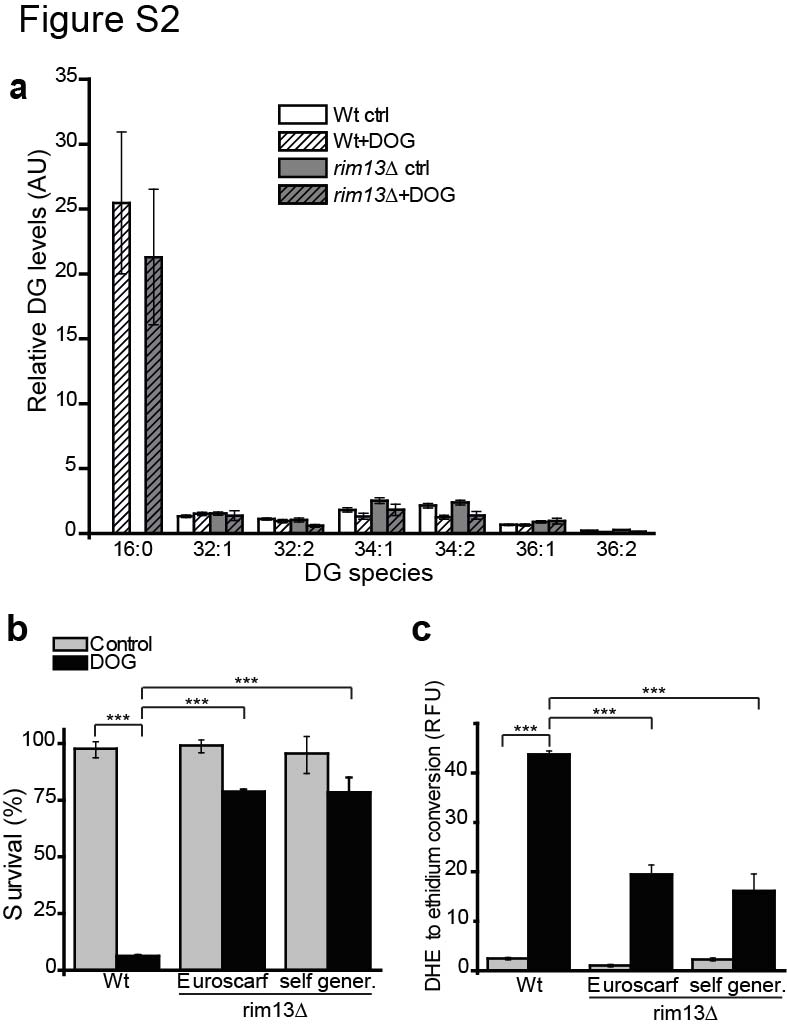


**Figure S2,** related to Fig. 5. (a) Profile of DG species with and without DOG treatment. The numbers on the x-axis indicate the cumulative number of carbon atoms (first number) and the cumulative number of double bonds of the DG acyl chains (second number after the colon) (b, c) Clonogenic survival (b) and ROS quantification (c) for self-generated rim13∆ strains. Error bars indicate standard error of the mean (SEM) and asterisks in the figures indicate significant differences, *p<0.05, **p<0.01, ***p<0.001, ****p<0.0001.


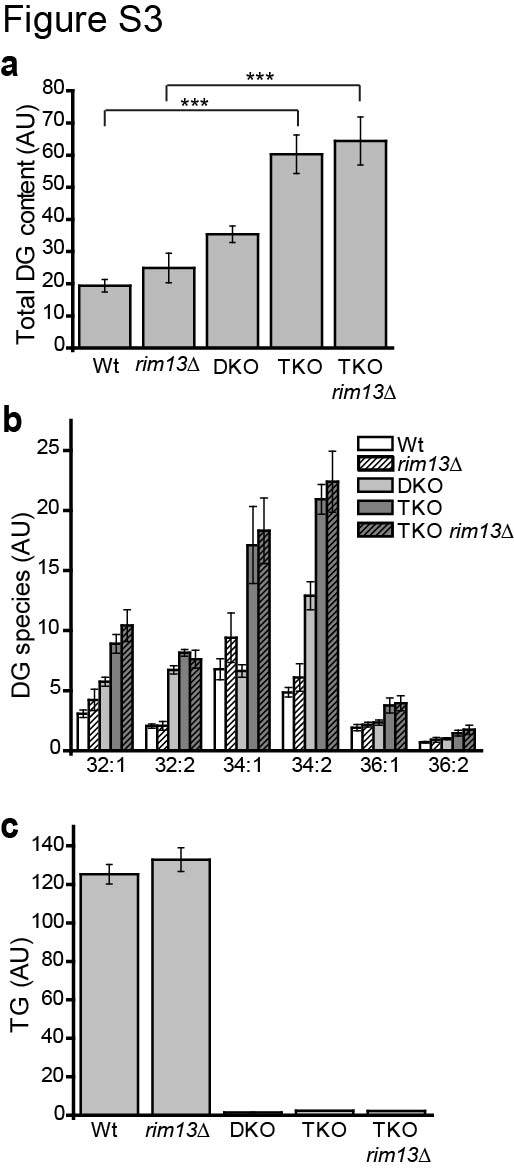


**Figure S3,** related to Fig. 5. (a-c) Additional mass spectrometry-assisted lipidomic profiles of DKO and TKO strains with and without additional *RIM13* deletion. (a) Total DG content (b) DG species The numbers on the x-axis indicate the cumulative number of carbon atoms (first number) and the cumulative number of double bonds of the DG acyl chains (second number after the colon) (c) Total TG. Error bars indicate standard error of the mean (SEM) and asterisks in the figures indicate significant differences, *p<0.05, **p<0.01, ***p<0.001, ****p<0.0001.


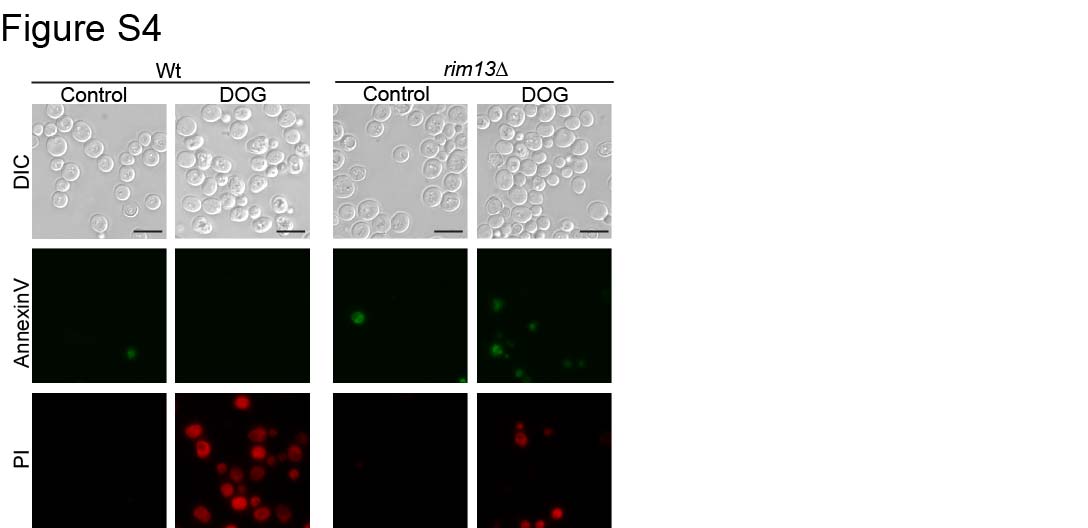


**Figure S4,** related to Fig. 5. Representative microscopy images of AnnexinV/ PI staining shown in Fig. 5i. Scale bar = 5 µm.


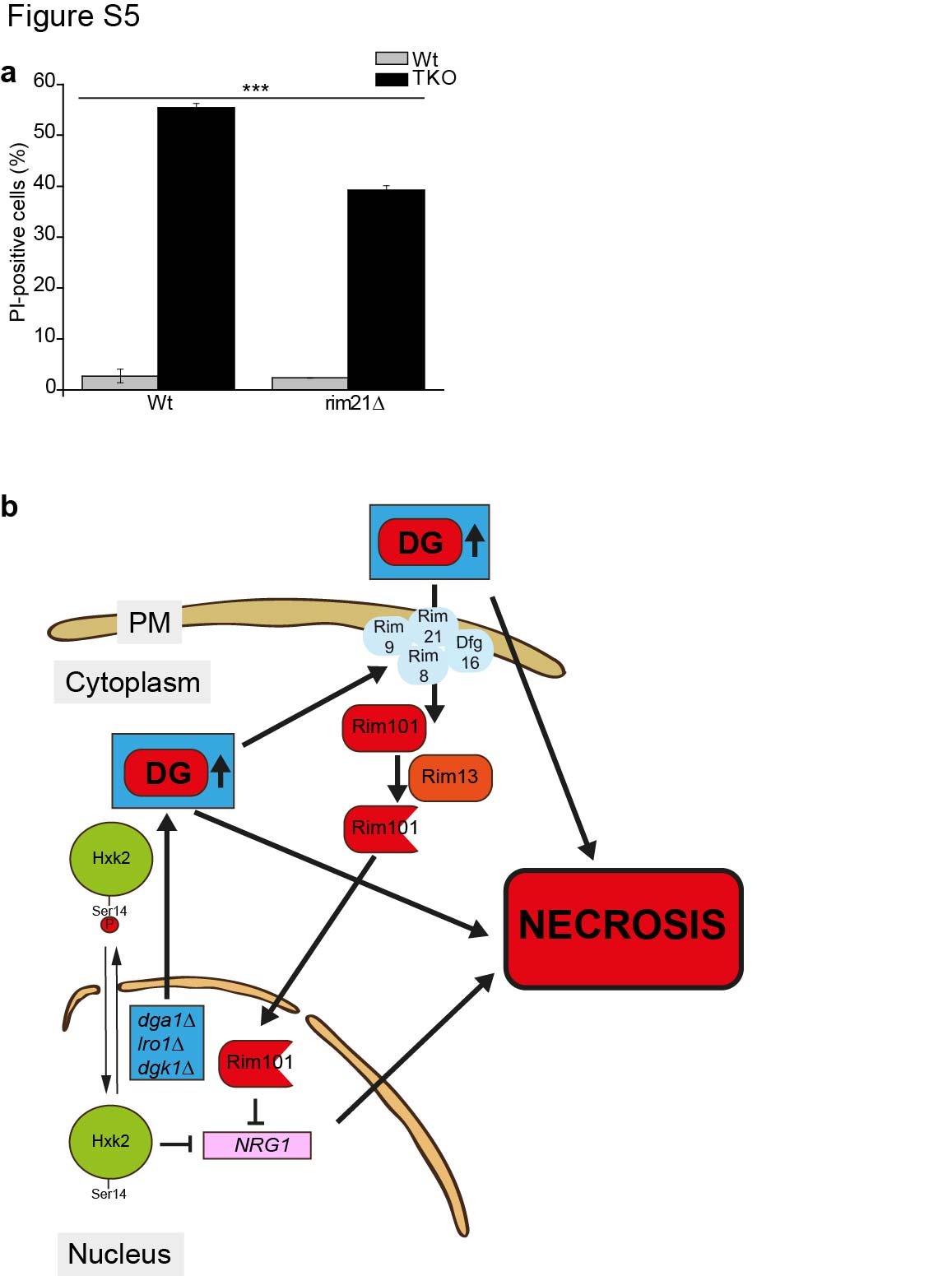


**Figure S5,** related to Fig. 6. Model for DAG-induced lipotoxicity in yeast

(a,) PI staining assessing cell death dependence on Rim21 in the TKO background. Error bars indicate standard error of the mean (SEM) and asterisks in the figures indicate significant differences of 2-way ANOVA analysis comparing TKO and *rim21* conditions as independent factors, *p<0.05, **p<0.01, ***p<0.001, ****p<0.0001. (b) Model of DG-induced cell death in yeast. Increased DG is sensed by the Rim sensor complex consisting of Rim21, Rim9, Dfg16 and Rim8. This further triggers Rim13 dependent cleavage of the transcriptional repressor Rim101. A transcriptional adaptation is integrated on the level of *NRG1*, which regulates survival and necrotic cell death in response to DG-increase.


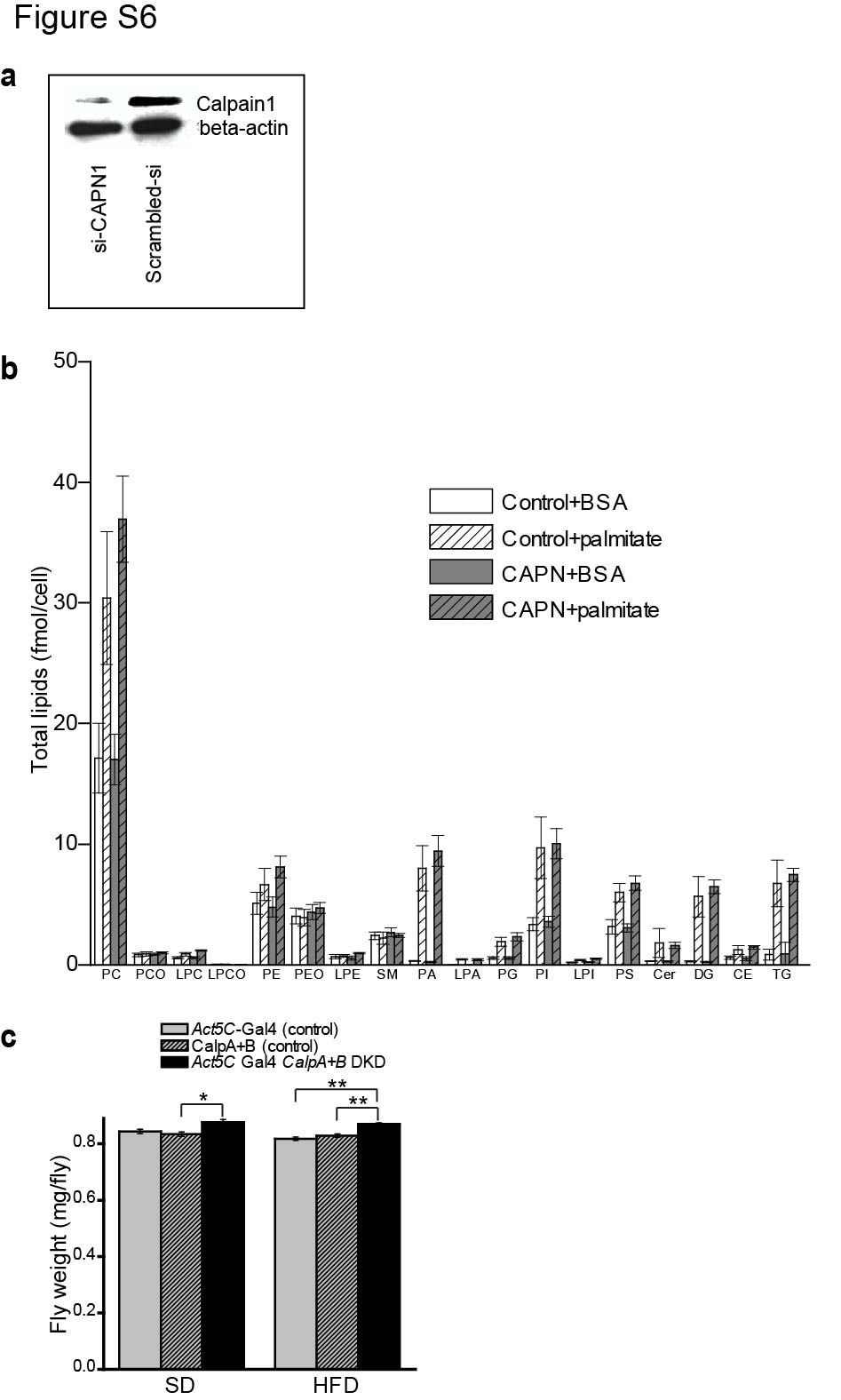


**Figure S6,** related to Fig. 7. (a) Immunoblot showing reduced Calpain1 abundance upon siRNA-mediated gene knock down in endothelial cells. (b) Lipidomic analysis of endothelial cells subjected to palmitate treatment and impact of siRNA-mediated Calpain1 knock down. Abbreviations: PC, phosphatidylcholine; PC-O, plasmenyl-phosphatidylcholine; LPC, lyso-phosphatidylcholine; LPC-O, plasmenyl-lyso-phosphatidylcholine; PE, phosphatidylethanolamine; PE-O, plasmenyl- phosphatidylethanolamine; LPE, lyso-phosphatidylethanolamine; SM, sphingomyelin; PA, phosphatidic acid; LPA, lyso-phosphatidic acid; PG, phosphatidylglycerol; PI, phosphatidylinositol; LPI, lyso-phosphatidylinositol; PS, phosphatidylserine; Cer, ceramide; DG, diglyceride; CE, cholesteryl ester; TG, triglyceride. (c) Weight of control and *CalpA+B* DKD flies was determined on standard diet (SD) and high fat diet (HFD) to control for potential differences in food uptake. Error bars indicate standard error of the mean (SEM) and asterisks in the figures indicate significant differences, *p<0.05, **p<0.01, ***p<0.001, ****p<0.0001.
